# Menstrual cycle does not impact the hypoxic ventilatory response and acute mountain sickness prediction

**DOI:** 10.1038/s41598-024-76404-y

**Published:** 2024-10-30

**Authors:** Tom Citherlet, Antoine Raberin, Giorgio Manferdelli, Vincent Pialoux, Grégoire P. Millet

**Affiliations:** 1 Institute of Sport Sciences, University of Lausanne, 1015 Lausanne, Switzerland; 2 Laboratoire Interuniversitaire de la Biologie et de la Motricité, Université Claude Bernard Lyon 1, Lyon, France

**Keywords:** Chemosensitivity, SpO_2_, ${\dot{\text{V}}\text{E}}$, Estradiol, Premenopausal, Female, Cardiovascular biology, Respiration, Physiology, Medical research

## Abstract

The relationship between the variations in ovarian hormones (i.e., estrogens and progesterone) and the hypoxic ventilatory response (HVR) remains unclear. HVR is a key adaptive mechanism to high altitude and has been proposed as a predictor for acute mountain sickness (AMS). This study aimed to explore the effects of hormonal changes across the menstrual cycle on HVR. Additionally, it assessed the predictive capacity of HVR for AMS and examined whether a particular menstrual phase could enhance its predictive accuracy. Thirteen eumenorrheic women performed a pure nitrogen breathing test near sea level, measuring HVR and cerebral oxygenation in early follicular, late follicular, and mid-luteal phases. Oxidative stress and ovarian hormone levels were also measured. AMS symptoms were evaluated after spending 14 h, including one overnight, at an altitude of 3,375 m. No differences in HVR, ventilation, peripheral oxygen saturation, or cerebral oxygenation were observed between the three menstrual cycle phases. Moreover, these parameters and the oxidative stress markers did not differ between the women with or without AMS (31% vs 69%), regardless of the menstrual cycle phase. In conclusion, ventilatory responses and cerebral oxygenation in normobaric hypoxia were consistent across the menstrual cycle. Furthermore, these parameters did not differentiate women with or without AMS.

## Introduction

Females are significantly under-represented in sports and exercise medicine research^1^ and their specific physiological responses to altitude need to be further investigated^2^. The relevance of the impact of ovarian hormones on altitude adaptations is also growing due to the increasing attractiveness of high-altitude environments (e.g., for work, training, or recreation). The menstrual cycle is a great model to evaluate the variations in ovarian hormone profiles^3^. During the early follicular phase (Fol1), both estradiol (E2) and progesterone (P4) levels are relatively low. Then, E2 increases significantly during the late follicular phase (Fol2), while P4 remains low. Finally, both E2 and P4 levels peak in the mid-luteal phase (Lut3) before declining.

Hypoxic exposure significantly impacts the respiratory and cardiovascular systems^4^. During the first hours of exposure, pulmonary ventilation and cardiac output increase in order to maintain tissue oxygenation^5^. While these systems are crucial for acclimatization, they are influenced by ovarian hormones^6,7^. For example, P4 stimulates ventilation (${\dot{\text{V}}\text{E}}$), and E2 potentiates its effect^6^. The hypoxic ventilatory response (HVR), which refers to the increase occurring when peripheral oxygen saturation (SpO_2_) decreases, has shown differences with the menstrual cycle. Some studies^3,8–10^ reported an increase in HVR during the luteal phase while others found no difference across the cycle^11,12^. These conflicting results suggest that further research is needed to clarify this relationship. Moreover, E2 increases cerebral blood flow^13^ and the latter has been linked to the pathogenesis of AMS in some studies^14^ (although this remains a topic of debate^15^). Additionally, it has been demonstrated that E2^8^ and P4^16^ reduce oxidative stress induced by chronic intermittent hypoxia in adult female rats. Higher levels of oxidative stress have also been found in the luteal phase of the menstrual cycle^17^. Altogether, these findings suggest that the menstrual cycle may modify the cerebrovascular, cardiorespiratory (e.g. HVR) and oxidative stress responses to hypoxia. This is supported by a large cross-sectional study^18^, which reported that physiological responses to hypoxic exercise were different between different the menstrual cycle phases. It is further reinforced by numerous sex differences on hypoxia tolerance, which have been reviewed recently^19^. Nevertheless, recent data challenge this, showing no link between the menstrual cycle, menopause, or exogenous P4 and AMS, emphasizing the need for further research^20^.

Tolerance to high altitude is typically evaluated through measurement of ${\dot{\text{V}}\text{E}}$, SpO_2_, HVR, and cerebral oxygenation at rest and/or during exercise in normobaric hypoxia^21,22^. HVR is thought to be beneficial at high altitude as it promotes adaptations to maintain oxygen availability^23^ and is used in the prediction of AMS^21^. However, it is unclear whether its predictive effectiveness is improved when ovarian hormone levels are lower (e.g., early follicular phase) and HVR potentially reduced. During this phase, it can be hypothesized that women would be more vulnerable to hypoxia, potentially revealing higher sensitivity to AMS.

Additionally, a relation between cerebral oxygenation and AMS has been reported^24^. This indicated that the analysis of acute brain adaptation to hypoxia may uncover pathophysiological processes that may also help to predict AMS^25^.

Increased oxidative stress is also a well-documented response to high-altitude exposure^26^. It has been shown to modulate the ventilatory acclimatization to hypoxia^27,28^. It is also implicated in the development of AMS, with higher malondialdehyde (MDA) levels and lower superoxide dismutase (SOD) and glutathione peroxidase (GPx) activities^26^. Evaluating these biomarkers before high-altitude exposure can provide further insights for AMS prediction^29^, but has never been tested. Altogether, hormonal changes during the menstrual cycle may affect cerebrovascular and cardiorespiratory responses to hypoxia, as well as oxidative stress, potentially influencing AMS predictability.

The present study aimed to assess whether the menstrual cycle-induced hormonal variations have an impact on HVR, cerebral oxygenation, and oxidative stress. Additionally, it aimed to determine the effectiveness of HVR, cerebral oxygenation, and oxidative stress in predicting AMS and to evaluate whether such prediction is enhanced by measuring it during a specific phase of the menstrual cycle. We hypothesized that the elevated hormonal levels at Fol2 and Lut3 may increase HVR, when compared to Fol1. We also hypothesized that AMS prediction would be more effective when HVR is measured during Fol1, due to the potentially increased vulnerability to hypoxia.

## Methods

### Participants

Thirteen eumenorrheic women (age 32 ± 8 years, weight 63.5 ± 9.6 kg, height 167 ± 10 cm, menstrual cycle length 27 ± 3 days, ferritin level 35 ± 25 ng/mL, serum iron 19 ± 18 µmol/L, C-reactive protein 1.7 ± 1.5 mg/L) participated in the study. They were required to be between 20 and 40 years old, healthy, non-smokers, with a body mass index under 30 kg/m^2^, and not pregnant. They should not have stayed overnight above 3000 m recently, have a history of iron deficiency, or engage in competitive swimming or breath-hold diving. Additionally, they needed to be free from cardiovascular, respiratory, or central nervous system disorders; and not taking β-blocker medications. They also had to have a natural menstrual cycle lasting 21 to 35 days without menstrual irregularities like amenorrhea, anovulation, or oligomenorrhea. In addition, they needed to have no hormonal contraceptive or hormone therapy use for the past three months.

### Experimental design

**Fig. 1:**
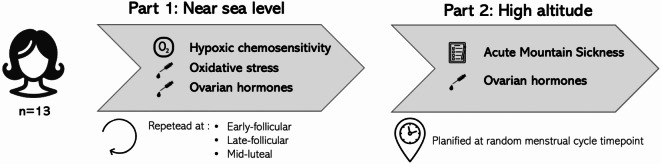
Study design.

The study was divided into two distinct parts (Fig. 1). During the first part, participants attended three different sessions corresponding to their personal Fol1, Fol2 and Lut3 phases. During each session, blood samples were collected at rest and participants performed a pure nitrogen breathing test (N_2_T). We employed the calendar-based method, tracking the first day of menstruation over the last three cycles to estimate the upcoming cycle length, which allowed us to improve accuracy in scheduling the sessions. The initial measurement order was random. This resulted in the measurement of Fol1 at 15 ± 11%, Fol2 at 44 ± 9%, and Lut3 at 76 ± 9% of their respective menstrual cycle lengths. Once participants completed the first part of the study, they were divided into two groups for the second part. Each group traveled on two successive weekends to the Torino Hut (3,375 m, Courmayeur, Italy), where they spent the night and were assessed for AMS.

The experimental protocol was approved by the Research Ethics Committee of the Canton Vaud (CER-VD 2022-00178) and conducted following the principles of the Declaration of Helsinki. All participants provided their written informed consent before their inclusion in the study.

### Data collection and analysis

During the first part of the study, participants had a 6 mL venous blood sample withdrawal from the antecubital vein. Samples were centrifuged at 3000G for 7:30 min (Sorvall ST8 centrifuge, Thermo Scientific, Tewksbury, MA, USA). The serum obtained was frozen at -80 °C and stored for later analyses. The serum levels of E2 and P4 were measured to ensure accurate identification of the menstrual phases. Additionally, to rule out any confounding factors affecting physiological responses to normobaric and hypobaric hypoxia, serum ferritin, ferrous ions, and C-reactive protein (CRP) were measured. Competitive enzyme-linked immunosorbent kits were used to quantify E2 levels (E2 ELISA kit, MyBioSource®, San Diego, CA, USA) and P4 levels (P4 ELISA kit, Abnova®, Taipei City, Taiwan). Sandwich enzyme-linked immunosorbent kits were employed to evaluate CRP concentrations (CRP ELISA kit, Abnova®, Taipei City, Taiwan), and ferritin concentration (Ferritin ELISA kit, Elabscience®, Houston, Texas, USA). A colorimetric assay kit was employed to measure ferrous iron concentrations (Ferrous iron colorimetric assay kit, Elabscience®, Houston, Texas, USA).

Oxidative stress markers were quantified including advanced oxidation protein products (AOPP), catalase, GPx, myeloperoxidase (MPO), nitrites, nitrates, total nitrite and nitrate (NOx), SOD, xanthine oxidase (XO), ferric-reducing antioxidant power (FRAP) and MDA.

Briefly, plasma AOPP concentration was measured in the presence of acetic acid (99–100%) and calculated using a chloramine-T standard solution, which absorbs at 340 nm with potassium iodide. XO activity was determined by measuring the appearance kinetics of the complex superoxide anion and nitrotetrazolium blue (NTB) by spectrophotometer at 560 nm for 10 min.

MPO activity was measured by a semi-quantitative immunoassay using stabilized human anti-MPO antibodies (MPO, Human, clone 266-6K1, HM2164, Hycult Biotech). The MPO/anti-MPO complex was detected by spectrophotometry after addition of a 3,3’,5,5’-tetramethylbenzidine solution with H_2_O_2_ as a chromogenic substrate. Nitrite levels were detected by fluorometry by using 2,3-diaminonaphtalene (DAN) that fixes nitrite and emits at 450 nm after excitation at 365 nm and computed with NO₂ standards. Total NOx levels were determined by reducing nitrate to nitrite using nitrate reductase, followed by measurement as described for nitrite. SOD activity was determined by the degree of inhibition of the reaction between superoxide produced by a hypoxanthine-xanthine oxidase system and NTB and corresponded to the difference between the slopes of the formation of formazan blue by the time of the blank and each sample. The activity of GPx was determined as the rate of oxidation of NADPH to NADP + in a cocktail solution containing glutathione reductase, NADPH and reduced glutathione using H_2_O_2_ as substrate at 340 nm for 5 min. GPx activity was measured as the slope of the NADPH extinction by time. Catalase activity was determined by measuring the kinetics of formaldehyde apparition formed by the reaction between methanol and H_2_O_2_, which is catalyzed by catalase. Formaldehyde was revealed by purpald solution, and its concentration was measured by spectrophotometry at 540 nm and computed using formaldehyde standards. MDA levels were quantified by spectrophotometry at 532 nm after heating the sample at 100 °C for 1 h with NaOH, 2-thiobarbituric acid, and HCl solutions. MDA concentration was calculated using 1,1,3,3-tetraethoxypropane as a standard.

FRAP was determined by the plasma’s ability to reduce ferric to ferrous iron, forming a complex with tripyridyltriazine at low pH, which was measured spectrophotometrically at 593 nm using ferrous iron standards.

**Fig. 2:**
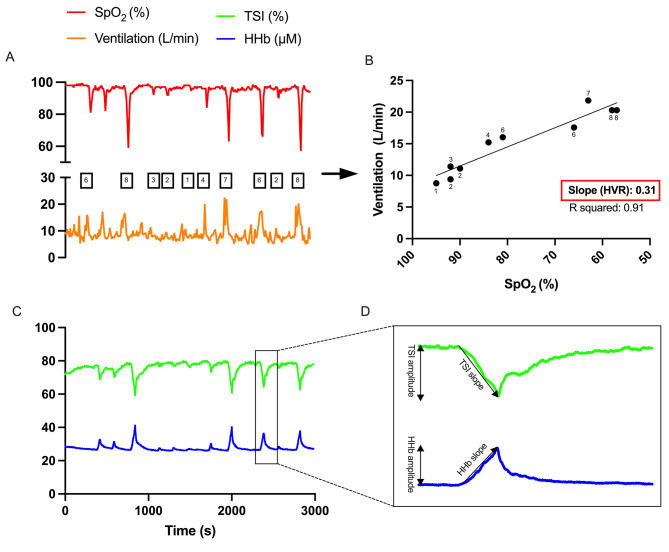
Measurements during the pure nitrogen breathing test in a typical participant. Panel (**A**) illustrates ventilation and peripheral oxygen saturation (SpO_2_) for each nitrogen exposure, with the number of breaths indicated within squares. The peak values are utilized for calculating the hypoxic ventilatory response (HVR) in Panel (**B**). Prefrontal cerebral oxygenation, indicated by total saturation index (TSI) and deoxyhemoglobin (HHb), is depicted in Panel (**C**), while Panel D displays the calculation of their slope and amplitude in a representative nitrogen exposure.

A N_2_T was used to measure HVR^22^. In a semi-recumbent position, participants began with a 3-min baseline measurement of ${\dot{\text{V}}\text{E}}$, SpO_2_, and cerebral oxygenation. Subsequently, they inhaled 100% nitrogen on 10 separate occasions, ranging from 1 to 8 breaths each, in a randomized order. Each nitrogen exposure was interspersed with periods of ambient air for at least 2 min. Room air-to-nitrogen transitions were accomplished by a 3-way valve. The HVR was determined by plotting the lowest finger SpO_2_ (WristOx® 3150 Nonin, Medical Inc., USA) against the highest ${\dot{\text{V}}\text{E}}$ (Quark metabolic cart, Cosmed, Rome, Italy) for each nitrogen exposure period (Fig. 2A). The absolute slope of this relation was indicative of HVR (Fig. 2B). We also calculated the ranges of ${\dot{\text{V}}\text{E}}$ and SpO_2_. SpO_2_ range is the mean of the individual changes from baseline to the lowest values while the ${\dot{\text{V}}\text{E}}$ range is the mean of the individual changes from baseline to the highest values.

Additionally, right prefrontal cerebral oxygenation was continuously recorded by near-infrared spectroscopy (NIRS; PortaLite, Artinis Medical Systems, Elst, The Netherlands). This device was placed on the prefrontal cortex. It comprises three dual-wavelength (760 and 850 nm) light transmitters positioned at intervals of 30, 35, and 40 mm from a single receiver optode. It was secured with a bandage to ensure stable contact and to reduce any external light interference. The tissue saturation index (TSI) and deoxygenated hemoglobin levels (HHb) were examined (Fig. 2C). We calculated their slopes and amplitudes (i.e., the magnitude of changes) in response to nitrogen exposures (Fig. 2D).

During the second part of the study, blood sampling was performed near sea level as in the first part to measure E2 and P4 levels. Participants were then transported to the Torino Hut by cable car, reaching it within 15–20 min. They were exposed to high altitude for a total of 14 h, including an overnight stay. AMS symptoms were evaluated 6 h post-arrival to altitude and the day after upon awakening using the revised Lake Louise Score^30^. Scores ≥ 3 were considered as AMS.

### Statistical analysis

A statistical power analysis was performed using G*Power 3.1 to determine the required sample size for this study based on previous chemosensitivity data^10^, where a large effect size (Cohen’s d = 1.16), with a significant increase (0.127) in hypoxic sensitivity from the follicular to the luteal phase has been reported. Consequently, for repeated measures ANOVA with three time points, a large effect (Cohen’s F = 0.04) was anticipated. The power analysis was set with a significance level (alpha) of 0.05 and a desired power (1-beta) of 0.80. It indicated that a sample size of 12 participants is necessary to achieve sufficient power to detect statistically significant differences.

Differences between the menstrual cycle phases were analyzed using a general linear model with repeated measures and corrected for multiple comparisons using Bonferroni correction. If Mauchly’s test indicated a violation of the assumption of sphericity, further corrections were applied to adjust the degrees of freedom. Shapiro–Wilk test, skewness, and kurtosis were used to check data distribution and no additional corrections were needed.

To compare the women prone to AMS (AMS+) or not (AMS−), a t-test for independent samples was conducted with the groups determined by AMS after 6 h at altitude. Levene’s test for equality of variances was employed to determine if the assumption of equal variances was violated. In case of violation, Welch’s t-test was used. Normality was checked using the Shapiro–Wilk test and, if necessary, the Mann–Whitney test was used to verify differences between AMS+ and AMS−.

All statistical analyses were performed using SPSS, version 26 (IBM Corp., Armonk, NY, USA). The significance level for all tests was set at P < 0.05.

## Results

**Fig. 3:**
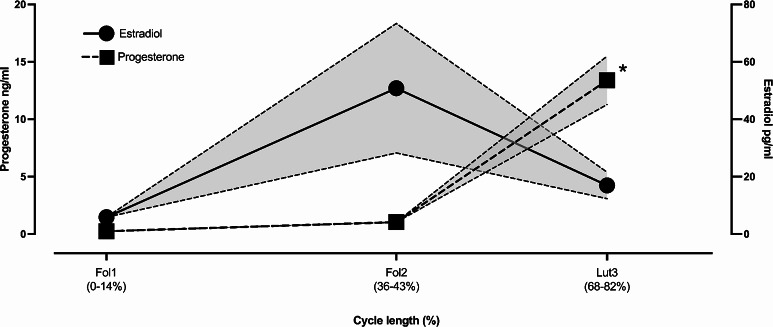
Ovarian hormones at early follicular (Fol1), late follicular (Fol2), and mid-luteal (Lut3) phases of the menstrual cycle. *, significantly higher than Fol1 and Fol2. Each symbol represents mean ± SEM.

As shown in Fig. 3, the E2 levels increased nine-fold from Fol1 (6 ± 2 pg/ml) to Fol2 (51 ± 81 pg/ml), and three-fold to Lut3 (17 ± 17 pg/ml). Meanwhile, the progesterone levels increased five-fold from Fol1 (0.2 ± 0.2 ng/ml) to Fol2 (1.0 ± 1.8 ng/ml), and 67-fold to Lut3 (13.4 ± 7.6 ng/ml).

**Fig. 4:**
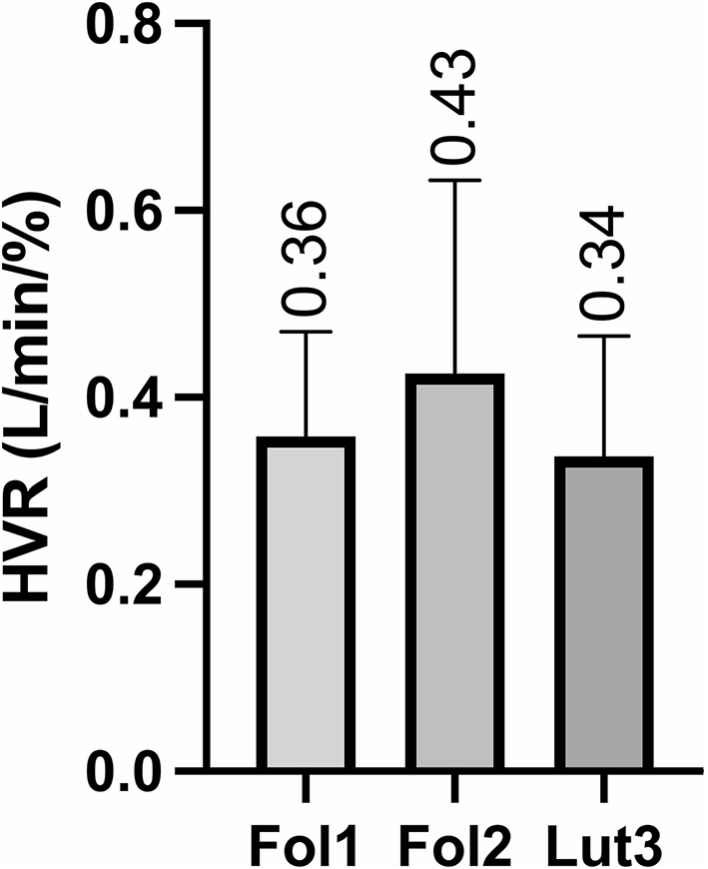
Hypoxic ventilatory response (HVR) at early follicular (Fol1), late follicular (Fol2), and mid-luteal (Lut3) phases of the menstrual cycle. Values are mean ± SD.

Despite these hormonal level variations, HVR did not change (P = 0.328) across the menstrual cycle (Fig. 4). The other parameters measured during N_2_T (i.e. ${\dot{\text{V}}\text{E}}$, SpO_2_, and cerebral oxygenation) did not vary either across the three menstrual cycle phases (Table 1). No significant difference was also observed for the oxidative stress parameters (Table 2), except for a higher FRAP value in Lut3 compared to Fol1 (P = 0.035).

**Table 1:** Pure nitrogen breathing test.

		*Fol1*	*Fol2*	*Lut3*
SpO_2_ in normoxia (%)	AMS+	93.8 ± 2.5	94.8 ± 1.7	94.5 ± 1.9
	AMS−	96.1 ± 1.9	96.4 ± 1.2	95.8 ± 1.2
	**Total**	**95.4 ± 2.2**	**95.9 ± 1.6**	**95.4 ± 1.5**
${\dot{\text{V}}\text{E}}$ in normoxia (L/min)	AMS+	9.5 ± 1.7	9.5 ± 1.2	9.8 ± 2.2
	AMS−	10.0 ± 0.9	9.9 ± 1.9	10.0 ± 1.2
	**Total**	**9.8 ± 1.2**	**9.8 ± 1.6**	**10.0 ± 1.5**
Hypoxic ventilatory response (L/min/%)	AMS+	-0.29 ± 0.06	-0.38 ± 0.25	-0.28 ± 0.06
	AMS−	-0.39 ± 0.12	-0.45 ± 0.20	-0.37 ± 0.15
	**Total**	**-0.36 ± 0.11**	**-0.43 ± 0.21**	**-0.34 ± 0.13**
Range ${\dot{\text{V}}\text{E}}$ (L/min)	AMS+	6.5 ± 1.8	6.3 ± 1.9	7.1 ± 0.8
	AMS−	9.0 ± 3.5	9.3 ± 3.9	8.9 ± 3.8
	**Total**	**8.2 ± 3.2**	**8.4 ± 3.6**	**8.2 ± 3.1**
Range SpO_2_ (%)	AMS+	18.0 ± 3.2	15.8 ± 5.3	18.5 ± 4.5
	AMS−	17.3 ± 6.3	18.4 ± 7.8	18.0 ± 6.0
	**Total**	**17.5 ± 5.3**	**17.6 ± 7.0**	**18.2 ± 5.3**
Total saturation index slope (%)	AMS+	-0.05 ± 0.03	-0.11 ± 0.08	-0.11 ± 0.03
	AMS−	-0.24 ± 0.15	-0.20 ± 0.12	-0.24 ± 0.18
	**Total**	**-0.18 ± 0.15**	**-0.17 ± 0.11**	**-0.19 ± 0.16**
Total saturation index Amplitude (%)	AMS+	-3.1 ± 1.3	-3.4 ± 1.2^†^	-5.6 ± 1.2
	AMS−	-6.2 ± 3.1	-6.7 ± 1.5	-6.5 ± 4.1
	**Total**	**-5.3 ± 3.0**	**-5.6 ± 2.1**	**-6.2 ± 3.2**
Deoxyhemoglobin slope (μM)	AMS+	0.09 ± 0.06	0.08 ± 0.06	0.08 ± 0.04
	AMS−	0.13 ± 0.07	0.09 ± 0.06	0.12 ± 0.03
	**Total**	**0.12 ± 0.07**	**0.09 ± 0.05**	**0.11 ± 0.04**
Deoxyhemoglobin amplitude (μM)	AMS+	3.3 ± 0.8	2.4 ± 1.0	4.4 ± 2.5
	AMS−	3.4 ± 0.6	3.7 ± 1.4	3.9 ± 0.7
	**Total**	**3.4 ± 0.6**	**3.2 ± 1.4**	**4.1 ± 1.3**

Fol1, early follicular phase; Fol2, late follicular phase; Lut3, mid-luteal phase; ${\dot{\text{V}}\text{E}}$, minute ventilation; SpO_2_, peripheral oxygen saturation. ^†^, significant difference between AMS+ and AMS−

Significant values are in bold.

**Table 2:** Oxidative stress markers.

		*Fol1*	*Fol2*	*Lut3*
Advanced oxidation protein products (μmol/L)	AMS+	420 ± 226	331 ± 83	262 ± 64
	AMS−	250 ± 127	270 ± 67	258 ± 67
	**Total**	**299 ± 171**	**289 ± 74**	**259 ± 64**
Catalase (μmol/L/min)	AMS+	11.2 ± 8	11.5 ± 7.9	10.6 ± 7.8
	AMS−	6.7 ± 5.9	7.7 ± 3	8.8 ± 4
	**Total**	**7.9 ± 6.6**	**8.9 ± 5**	**9.4 ± 5.1**
Ferric reducing antioxidant power (μmol/L)	AMS+	1184 ± 214	1262 ± 406	1171 ± 232
	AMS−	1080 ± 260	1191 ± 320	1294 ± 253
	**Total**	**1110 ± 245**	**1213 ± 332**	1256 ± 244*
Glutathione peroxidase (μmol/L/min)	AMS+	4.6 ± 0.7	5.3 ± 0.5	4.7 ± 1.0
	AMS−	4.3 ± 1.0	4.5 ± 1.0	4.8 ± 0.6
	**Total**	**4.4 ± 0.9**	**4.7 ± 0.9**	**4.8 ± 0.7**
Malondialdehyde (μmol/L)	AMS+	2.3 ± 1.5	2.8 ± 0.3	2.7 ± 1.7
	AMS−	2.2 ± 0.6	3.4 ± 3.3	2.1 ± 0.9
	**Total**	**2.2 ± 0.9**	**3.2 ± 2.7**	**2.3 ± 1.1**
Myeloperoxidase (μmol/L/min)	AMS+	129 ± 72	71 ± 25	164 ± 187
	AMS−	116 ± 74	76 ± 25	78 ± 57
	**Total**	**120 ± 71**	**75 ± 24**	**105 ± 112**
Nitrites (μmol/mL)	AMS+	20 ± 15	21 ± 16	20 ± 7
	AMS−	16 ± 8	18 ± 14	16 ± 13
	**Total**	**17 ± 10**	**19 ± 14**	**17 ± 12**
Nitrates (μmol/mL)	AMS+	40 ± 14	42 ± 17	44 ± 22
	AMS−	38 ± 13	45 ± 19	46 ± 19
	**Total**	**39 ± 12**	**44 ± 18**	**45 ± 19**
Total nitrite and nitrate (μmol/mL)	AMS+	20 ± 3	21 ± 2	24 ± 17
	AMS−	22 ± 6	27 ± 11	29 ± 9
	**Total**	**21 ± 6**	**25 ± 9**	**28 ± 11**
Total superoxide dismutase (μmol/L/min)	AMS+	11 ± 3	13 ± 6	18 ± 14
	AMS−	16 ± 6	13 ± 5	17 ± 9
	**Total**	**14 ± 6**	**13 ± 5**	**17 ± 10**
Xanthine oxidase (μmol/L/min)	AMS+	7 ± 2	9 ± 5	9 ± 2
	AMS−	8 ± 4	7 ± 3	8 ± 2
	**Total**	**8 ± 3**	**8 ± 4**	**8 ± 2**

Fol1, early follicular phase; Fol2, late follicular phase; Lut3, mid luteal phase. *, higher than Fol1.

Pooled data for AMS+ and AMS- are in bold.

AMS was observed in 31% of participants after 6 h, while it decreased to 15% after 14 h, including an overnight. The AMS+ group did not exhibit any difference compared to the AMS− group in the parameters measured during N_2_T (Table 1), except for a lower TSI amplitude at Fol2 (P = 0.012). There was no difference in the oxidative stress markers (Table 2), E2, and P4 hormone levels measured on the day of ascent (Table 3).

**Table 3:** Ovarian hormones before ascending to high altitude.

Estradiol (pg/mL)	AMS+	9.63 ± 4.07
	AMS−	17.24 ± 15.52
	**Total**	**14.90 ± 13.34**
Progesterone (ng/mL)	AMS+	5.59 ± 8.96
	AMS−	4.75 ± 6.73
	**Total**	**5.01 ± 7.11**

AMS, acute mountain sickness.

Pooled data for AMS+ and AMS- are in bold.

## Discussion

This study aimed to investigate the influence of hormonal variations during the menstrual cycle on HVR, cerebral oxygenation, and oxidative stress. Additionally, it aimed to evaluate the predictive value of these parameters for AMS and determine if accuracy improves when measured during different menstrual cycle phases. We observed no significant differences in HVR, cerebral oxygenation, and oxidative stress across the menstrual cycle. Additionally, none of these parameters differed between AMS+ and AMS−.

(i) HVR remained constant and oxidative stress showed minimal variation across the menstrual cycle.

No difference was observed throughout the menstrual cycle on HVR, or any other responses measured during the N_2_T. These results are consistent with previous studies reporting similar HVR across menstrual cycle in healthy women^11,12^. However, there are also contradictory findings reporting an increased HVR during the luteal phase^10,18,31,32^. Of interest, HVR has also been reported to increase with pregnancy^33,34^, hormonal treatment^12^, and decrease with ovariectomy^35^. These findings reinforce the assumption of sex hormones influence. The underlying mechanism may rely on progesterone enhancing HVR by raising the carotid body’s responsiveness to hypoxia. Additionally, when combined with estrogen, progesterone may also modulate the central processing of signals from the carotid body^33^. However, despite hormonal level variations during the menstrual cycle, we observed no differences in HVR and other parameters. These results might be influenced by several factors such as the low E2 levels observed. Indeed, E2 reference intervals are 31–771 pmol/L during Fol1, 104–1742 pmol/L during the Fol2, 151–1941 pmol/L during Lut3^36^. In contrast, the E2 levels in this study were 6 ± 2 pg/ml (22 ± 7 pmol/L) at Fol1, 51 ± 81 pg/ml (187 ± 297 pmol/L) at Fol2, and 17 ± 17 pg/ml (62 ± 62 pmol/L) at Lut3. Despite falling within the reference range for Fol1 and Fol2, the overall E2 levels remained notably low. Sex hormones may induce HVR changes that are not detectable, as in the present study. The differences with previous reports may also stem from the HVR protocols, such as duration, degree of hypoxia, participant position, and isocapnia versus poikilocapnia. Additionally, variations in methods to identify the menstrual cycle phases, or the number of phases compared could contribute to these differences. For example, HVR is reduced by 52% when measured in the supine position^37^ which could attenuate its variations across the menstrual cycle. In the present study, a semi-recumbent position was used. This differs from previous studies that employed seated, reclined, or supine positions with the trunk and head elevated. Also, a poikilocapnic protocol was used as it best reflects the physiological changes occurring at altitude. However, an isocapnic protocol could yield different results due to the potent ventilatory stimulating effect of CO_2_.

Similarly, SpO_2_ did not vary with the menstrual cycle, which is in line with previous measurements at rest^38^. However, during exercise in hypoxia, findings differ, with some studies reporting no changes in SpO_2_^39,40^ vs higher levels at Lut3^11,18^. Different mechanisms underlying the increased SpO_2_ at Lut3 have been proposed such as enhanced HVR^18^ and improved pulmonary gas exchange^11^. Enhanced lung diffusion has also been observed^41^. Similarly, cerebral oxygenation did not vary with the menstrual cycle despite previous research showing vasodilation in the right hemisphere^42^. This vasodilation could be explained by the ventilatory stimulant effect of ovarian hormones observed at Lut3^43^ which could lower alveolar CO_2_ pressure, a cerebral vasodilator^44^.

It has been suggested that E2 has antioxidant properties due to the presence of phenolic group^17^. In this study, FRAP was significantly higher at Lut3 than at Fol1 but no other oxidative stress markers showed a significant difference. This suggests that fluctuations in E2 and P4 during the menstrual cycle have a minimal impact on oxidative stress. These findings corroborate previous reports^45^ while others found the maximal oxidative stress value near the E2 peak^46^ and during the luteal phase^17^. Differences between studies may arise from the methodologies used and the specific biomarkers analyzed.

(ii) HVR, cerebral oxygenation, oxidative stress, and hormonal levels did not predict the occurrence of AMS.

AMS occurred in about one-third of the participants. This aligns with the known rate at similar altitude^47^. Nevertheless, neither HVR, nor other N_2_T parameters nor oxidative stress markers were different between AMS+ and AMS−. The usefulness of HVR measurement to predict AMS remains debated. Accurate prediction of AMS through HVR measurement in normobaric hypoxia has been reported in some studies^48–51^, but not all^52–54^. Once again, these discrepancies may originate from the methods to measure HVR^55^. In many studies, the hypoxic challenge was brief, yet extended exposure, such as 20–30 min^56^ significantly improved the accuracy of AMS prediction to 86%. Richalet et al.^21^ also demonstrated an integrated method by merging HVR in a scoring system, the SHAI score, and considering HVR at exercise. The SHAI score, where HVR is the most influential factor^57^, yielded a positive predictive value of 29%. It should be considered that individuals identified as at higher risk of AMS may engage in preventive behaviors, which could impact the positive predictive value. Furthermore, the test demonstrated a negative predictive value of 81%, highlighting its accuracy^21^.

In our study, most TSI variables did not differ between AMS+ and AMS− groups, except for a reduced TSI amplitude at Fol2 in AMS+ subjects. These results partially align with a previous study^24^ that investigated resting and exercising prefrontal cerebral oxygenation in hypoxia and its association with AMS occurrence. Similarly to our results, they reported no significant difference in cerebral oxygenation at rest between AMS+ and AMS− subgroups. However, they observed decreased TSI during exercise in AMS+ individuals, contrasting with our findings. This also challenges the hypothesis that individuals with marked exercise-induced cerebral deoxygenation are at a higher risk of severe AMS symptoms^25^. Our findings mostly corroborate research suggesting the absence of association between cerebral blood flow and AMS^58–60^, despite some studies presenting opposing evidence^14,61^. It is, however, important to keep in mind that TSI primarily measures blood oxygenation rather than blood flow.

No differences in oxidative stress markers measured before ascent were observed between AMS+ and AMS− individuals. This suggests that baseline resting normoxic levels of these biomarkers may not be predictive of AMS occurrence. Instead, the dynamic changes in these biomarkers following hypoxic tests or high-altitude exposure might offer a more accurate prediction and understanding of AMS susceptibility. Notably, the increased HVR observed after hypoxic exposure is associated with increased oxidative stress^27^. Additionally, it has been shown that MDA levels increase in response to hypoxic exercise specifically in individuals predisposed to high-altitude pulmonary disease^62^.

Similarly, the absence of differences in the ovarian hormone levels between AMS+ and AMS− subgroups reinforces the assumption that hormonal levels have a limited—if not negligible—impact on susceptibility to AMS occurrence. In accordance, previous findings^63^ reported comparable ${\dot{\text{V}}\text{E}}$ and HVR at altitude in participants in the follicular compared to the luteal phase. Other findings also reported the absence of an association between AMS and the menstrual cycle phase, menopause, and exogenous P4^20^.

## Strengths and limitations

This study benefits from a comprehensive investigation into the relationship between the menstrual cycle and physiological responses to hypoxia. It examines multiple well-established (e.g., HVR, SpO_2_, ${\dot{\text{V}}\text{E}}$) and novel (e.g., cerebral oxygenation) parameters. Additionally, iron status, known to influence many physiological outcomes^64^, has also been evaluated. Moreover, rigorous participant selection criteria were applied to maintain internal validity. Furthermore, our study design incorporates essential controls, notably the use of hormonal levels as a reference method for cycle phase control.

Despite its strengths, this study is subject to several limitations. Accurately timing tests to coincide with peak hormonal phases is complicated by individual variations in cycle length and rapid hormone fluctuations. Despite controlling for hormonal levels, it is not possible to know whether the timing could have been improved and ultimately influenced the results. Also, significant individual differences exist in hormonal levels and profiles. Only P4 demonstrated significantly higher levels during Lut3 compared to Fol1 and Fol2 (P < 0.001), with no other significant variations in P4 or E2 levels. This suggests that Fol2 may not have been accurately captured, as E2 levels did not differ from Fol1. As described earlier, E2 levels were also notably low compared to reference values. This can explain the absence of differences in E2 between phases. Additionally, the N_2_T accuracy may be influenced by random breathing variations and the often lower desaturation in participants with low breathing frequencies. The test is also performed with short hypoxic exposures in normobaric hypoxia and resting conditions. However, it is important to note that AMS manifests over several hours and HVR varies during hypoxic exposure. This study measured the acute ventilatory response to hypoxia occurring within minutes and does not consider the hypoxic ventilatory decline that happens subsequently. It is also worth noting that normobaric hypoxia may induce different physiological responses from hypobaric hypoxia, as it has been a topic of debate^65^. Moreover, measurements were not performed during exercise while it is an additional stress that could reveal differences that are not visible at rest. This hypothesis is supported by the absence of difference in SpO_2_ at rest across the menstrual cycle, contrasted by differences observed during exercise in the studies mentioned above. Finally, this study was conducted at 3,375 m, which may not apply to higher altitudes.

## Conclusion

In conclusion, this study reports that hormonal fluctuations throughout the menstrual cycle do not affect HVR. Furthermore, no reliable predictors of AMS were found among the N_2_T variables, cerebral oxygenation, or oxidative stress markers, independently of the menstrual cycle phases.

## Data Availability

Data are available from the corresponding author upon request.
